# Regulation of p53 in NIH3T3 mouse fibroblasts following hyperosmotic stress

**DOI:** 10.14814/phy2.12412

**Published:** 2015-06-08

**Authors:** Ian Henry Lambert, Maria Stine Enghoff, Marie-Luise Brandi, Else Kay Hoffmann

**Affiliations:** Department of Biology, Section of Cellular and Developmental Biology, University of CopenhagenCopenhagen, Denmark

**Keywords:** Apoptosis, MDM2, p38

## Abstract

The aim of this project was to analyze the regulation of p53 expression in NIH3T3 fibroblasts under the influence of increasing hyperosmotic stress. Expression of p53 showed a biphasic response pattern in NIH3T3 cells under increasing osmotic stress (337 mOsm to 737 mOsm) with a maximum at 587 mOsm. Under isotonic conditions p53 expression increased after addition of the proteasome inhibitor MG132 indicating that cellular p53 levels in unperturbed cells is kept low by proteasomal degradation. However, under hypertonic conditions p53 synthesis as well as p53 degradation were significantly reduced and it is demonstrated that the increase in p53 expression observed when tonicity is increased from 337 to 587 mOsm reflects that degradation is more inhibited than synthesis, whereas the decrease in p53 expression at higher tonicities reflects that synthesis is more inhibited than degradation. The activity of the p53 regulating proteins p38 MAP kinase and the ubiquitin ligase MDM2 were studied as a function of increasing osmolarity. MDM2 protein expression was unchanged at all osmolarities, whereas MDM2 phosphorylation (Ser^166^) increased at osmolarities up to 537 mOsm and remained constant at higher osmolarities. Phosphorylation of p38 increased at osmolarities up to 687 mOsm which correlated with an increased phosphorylation of p53 (Ser^15^) and the decreased p53 degradation. Caspase-3 activity increased gradually with hypertonicity and at 737 mOsm both Caspase-3 activity and annexin V binding are high even though p53 expression and activity are low, indicating that initiation of apoptosis under severe hypertonic conditions is not strictly controlled by p53.

## Introduction

p53, which was discovered more than 30 years ago, is the most widely studied tumor suppressor (Levine and Oren 2009) and more than 50% of human cancers possess mutation in p53 (Levine and Oren 2009). p53 initiates cell cycle arrest, apoptosis, senescence, or autophagy in response to certain stress factors, for example, UV-light exposure, hypoxia, ribosomal stress, and osmotic stress (Kruse and Gu 2009; Elias et al. 2014).

The ubiquitin ligase MDM2 (murine double minute 2) is a major player in p53 regulation as it promotes proteasomal p53 degradation (Elias et al. 2014). Under normal unstressed conditions p53 controls its own degradation by promoting *MDM2* transcription (Moll and Petrenko 2003). During cell stress p53 has to be stabilized and relieved from the MDM2 interaction in order to activate the cells stress response. Phosphorylation of p53 is a known modification and it has been demonstrated that phosphorylation of p53 at Ser^15^ by the serine/threonine protein kinase ATM (ataxia telangiectasia mutated) and the p38 MAP (microtubule-associated protein) kinase (She et al. 2000) or at Ser^20^ by ATM blocks the p53 MDM2 interaction and hence p53 ubiquitination (Elias et al. 2014). Like p53 MDM2 can be regulated by posttranslational modifications, that is, phosphorylation of MDM2 Ser^395^ inhibits the activity of MDM2 whereas phosphorylation of MDM2 Ser^166^ activates MDM2 translocation to the nucleus and its E3-ligase activity (Meek and Knippschild 2003). In addition to proteasomal degradation the p53 protein level is also regulated on a translational level by the ribosomal protein L26 and nucleolin, which increases and prevents p53 translation, respectively (Takagi et al. 2005).

Hypertonic stress causes cell shrinkage and leads to activation of a signaling cascade, which involves p53 and ends with apoptosis (Hoffmann et al. 2009). Induction of apoptosis via the intrinsic pathway involves p53 interaction with multidomain members of the Bcl-2 family, permeabilization of the outer mitochondrial membrane, release of cytochrome c and subsequently activation of caspase-3 (Vaseva and Moll 2009). In NIH3T3 mouse fibroblasts we have shown that the GTP binding protein Rac is activated when cells are transferred from isotonic (337 mOsm) to hypertonic (687 mOsm) and that Rac subsequently activates p38, which, as mentioned above, stabilizes p53 via phosphorylation at Ser^15^ (Friis et al. 2005). Consequently an increase in the level of p53 is associated to an increase in caspase-3 activity.

The goal of this project was to observe regulation of p53 expression in NIH3T3 fibroblasts under influence of increasing hyperosmotic stress. We report that the level of p53 shows a biphasic activation of p53 at increasing osmolarities and investigate the effect of increasing hypertonic stress on the proteins which seem to have a role in p53 regulation like p38 and MDM2. Additionally proteasomal degradation and the translational regulation of p53 were studied during the project. Finally we want to see if increase in caspase-3 activity with increasing osmolarity correlates with the p53 expression pattern.

## Materials and Methods

### Cell culture

Swiss NIH3T3 fibroblasts, derived from mouse embryonic fibroblast cells, were cultured in T175 flasks with Dulbecco's Modified Eagles Medium (DMEM) supplemented with 10% (v/v) heat-inactivated fetal bovine serum and 1% penicillin/streptomycin mix. The cells were kept in the incubator at 37°C, 95% humidity and 5% CO_2_. The cell culture was passaged every 3–4 days by trypsination (0.5%). Only passages 10–30 were used.

### Chemicals and antibodies

Antibiotics (penicillin, streptomycin), DMEM (Gibco, high glucose, L-glutamine), fetal calf serum (Gibco), and trypsin (10×, Gibco) were from Invitrogen (Life Technology, Waltham, MA, USA). Unless otherwise stated, chemicals were purchased from Sigma Aldrich (St. Louis, MO) or Mallinckrodt Baker B.V. (Deventer, NL). The proteasome inhibitor MG 132 was prepared as a 10 mmol/L stock-solution in DMSO is used. Primary antibodies: p53 (1C12) and phospho-p53 (Ser15) Abs (53 kDa; Cell Signaling, Essex, MA, USA, Mouse IgG, 1:500), p38 and phospho-p38 MAP kinase Abs (Thr180/Tyr182) (38 kDa; Cell Signaling, Rabbit IgG, 1:200), MDM2 Ab (90 kDa, R&D Systems, affinity-purified Rabbit IgG, 1:500), phosphorylated MDM2 Ab (Ser166) (pMDM2, Cell Signaling, Rabbit, IgG 1:500), and histone H3 Ab (FL-136) (Santa Cruz Biotechnology; Finnell Street, Dallas, TX, USA, Rabbit IgG, 1:250) were all prepared in NaAzid free blocking buffer. Stabilized peroxidase conjugated secondary antibodies: Goat Anti-Mouse (Thermo Fisher Scientific, Wyman Street, Waltham, MA, USA, 1:600) and Goat Anti-Rabbit (Thermo Scientific; 1:600) were prepared in NaAzid free blocking buffer. Luminol and enhancer for western blotting were from Thermo Scientific.

### Media

The phosphate-buffered saline (PBS) contained 137 mmol/L NaCl, 2.6 mmol/L KCl, 6.5 mmol/L Na_2_HPO_4_, and 1.5 mmol/L KH_2_PO_4_. Hypertonic DMEM was prepared by adding a calculated amount of 2.5 mol/L NaCl stock solution to the growth medium. Osmolarity of the solutions was verified by freezing point depression (Knauer Osmometer, Berlin, Germany). For the proteasome inhibition experiments, 330, 587, and 737 mOsm media were used and the proteasome inhibitor MG132, dissolved in DMSO was diluted 1:1000 in the media.

### Cell lysates and western blotting

1 × 10^6^ cells were plated in nine petri dishes (diameter 10 cm) about 24 h before the experiment to ensure an 80% confluence. The growth medium was removed by suction and cells incubated at 37°C for 1, 2, 3, 4 h (time traces), 2 h (Caspase-3 activity), or 4.5 h (western blotting, QPCR) in either isotonic (330 mOsm) or hypertonic (437, 500, 537, 587, 600, 637, 687, and 737 mOsm) DMEM medium. Cells were subsequently washed with ice-cold PBS, the PBS was removed by suction and the cells lysed in preheated (95°C) lysis buffer containing 10 mmol/L Tris-HCl, 1% SDS, 20 mmol/L EDTA plus 0.5 mmol/L of the protease inhibitor Na_3_VO_4_. A rubber police man was used to scrape off the cells before the homogenate was transferred to an Eppendorf tube and heated again at 95°C for 5 min. To ensure complete cell lysis the lysate was sonicated (medium level 4) for 20 sec. Finally, samples were heated again at 95°C for 3 min and spun cell debris was spun down at 20,000 *g* at 4°C for 5 min. The supernatant, containing the protein of interest, was transferred to a new tube and protein quantified by a BioRad Protein Assay (Alfred Nobel Drive, Hercules, CA, USA), using bovine serum albumin (Pierce) as standard and optical detection at 600 nm (GeneQuant™, GE Healthcare, Bio-Sciences, Pittsburg, PA, USA).

Lysate samples (30 *μ*g protein) were separated and analyzed using Sodium Dodecyl Sulfate (SDS) PAGE and western blot as previously described (Friis et al. 2005). Briefly, samples were separated on NuPAGETM (Invitrogen) Novex 10% Bis-Tris gels, using NuPAGE 3-Morpholinopropanesulfonic acid (Mops) SDS running buffer and transferred to Whatman protran BA83 nitrocellulose transfer membranes (GE Healthcare) using a XCell II BlotModule (Invitrogen). Staining with 1% Ponceau S Red (Sigma) was used to verify/evaluate protein transfer before membranes were washed with TBST (10 mmol/L Tris-HCl, pH 7.4, 120 mmol/L NaCl, and 0.1% Tween 20) and incubated/blocked for 2 h at room temperature or overnight at 4°C in TBST containing 5% nonfat dry milk to prevent unspecific protein binding. Membranes were incubated with primary antibodies for 2 h at room temperature or overnight at 4°C using primary antibodies diluted in blocking buffer without NaAzid. After incubation with the primary antibody membranes were washed in TBST and subsequently incubated with peroxidase-conjugated secondary antibodies diluted in blocking buffer without NaAzid. The membranes were incubated at room temperature for 1 h, washed in TBST, added a luminol/enhancer solution 1:1, where after protein bands were detected by their chemo luminescence in Kodak Image Station 2000 MM. Protein bands were quantified by the UN-SCAN-IT gel 6.1 program.

### Real-time quantitative polymerase chain reaction (RT-qPCR)

RT-qPCR was performed to quantify the mRNA accumulation/gene expression of p53 and *β*-actin (reference gen). RNA was isolated from cells grown to 90% confluence in petri dishes, using lysis buffer containing guanidine thiocyanate, plus *β*-mercapto-ethanol and a GenEluteTM Mammalian Total RNA Kit (Sigma Aldrich). Reverse transcription was performed on 1 *μ*g RNA aliquots (determined from the 260 nm/280 nm absorbance ratio) and using an AffinityScript QPCRTM cDNA synthesis kit (Agilent Technologies, Stratagene Products Division, Santa Clara, CA, USA), a Mastercycler® (Eppendorf) and the temperature scheme: 5 min at 65°C (RNA denaturation), few minutes on ice (oligo(dT) primers annealing to poly(A)-tails), 15 min at 42°C (cDNA synthesis) and 5 min at 95°C (termination of the reaction). To quantify cDNA (mRNA) accumulation we used the Brilliant II SYBR® Green QPCR master mix (Stratagene) plus the following primers (100 pmol/*μ*L) and TaqMan probes conjugated with 5′fluorescein:

p53 forward primer: 5′-ATC TGG AAG ACA GGC AGA C-3′
p53 reverse primer: 5′-CCA TGC AGG AGC TAT TAC AC-3′
p53 probe: 5′-CCG GCT CTG AGT ATA CCA CCA TCC ACT A-3′
*β*-actin reverse primer: 5′-GGA TGC CAC AGG ATT CCA AAC-3′
*β*-actin forward primer: 5′-AGA GCT ATG AGC TGC CTG AC-3′
*β*-actin probe: 5′-CCC TGA GGC TCT TTT CCA GCC TTC CTT CTT-3′

### Caspase-3 activity assay – apoptosis

Caspase-3 activity in NIH3T3 cells was measured as previously described using a ApoTargetTM Caspase-3/CPP32 Colorimetric assay (Protease BioSource International) and determination of protease activity from the cleavage of the peptide substrate acetyl-Asp-Glu-Val-Asp p-nitroanilide (Ac-DEVD-pNA) to p-nitroanilide (pNA), measured as shift in absorbance at 405 nm (microplate reader, Bmg LabTechnologies, Offenburg, Germany (Tastesen et al. 2010). Apoptotic and necrotic cells were detected by confocal laser scanning (Leica SP5X) using a commercial kit (Life Technology) and Annexin V-488 (Life Technology) binding (apoptosis) and propidium iodide staining (necrosis).

### Viability and cell count

Cells, grown in Greiner Bio-one 12-well cell culture plates (500/000 cells per well) were exposed to isotonic and hypertonic conditions for 2 or 4½ h and subsequently detached by trypsination. Trypsin was neutralized by addition of medium. Cell necrosis was determined in a Nucleo Counter NC-200 using the Via1-CasetteTM system (Chemotec, Davis, CA, USA), that is, concomitant staining with acridine orange for determination of total number of cells and DAPI for the number of permeable, necrotic cells. Number of necrotic cells is given relative to the total number of cells.

### Statistical analysis

Data are presented either as individual experiments, that are representative of at least three independent sets of experiments or as mean values ± standard error of the mean (SEM). Statistical significance was estimated by ANOVA. For all statistical evaluations, *P* values < 0.05 were taken to indicate a significant difference.

## Results

### p53 expression and phosphorylation as function of increasing osmotic stress

**Figure 1:**
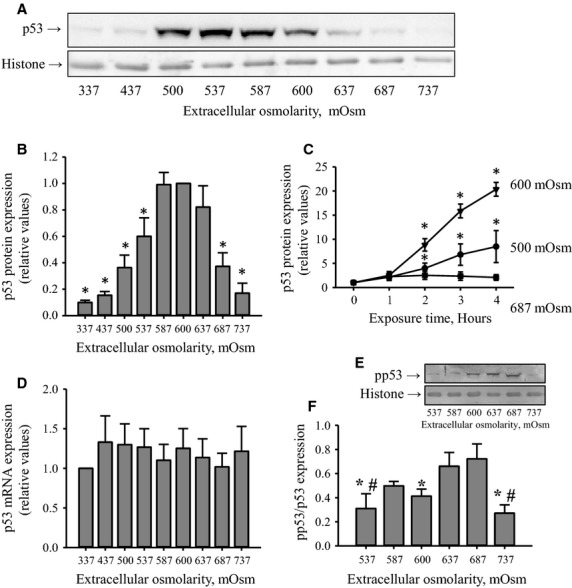
p53 protein expression, phosphorylation and p53 mRNA accumulation in NIH3T3 cells after exposure to isotonic and increasing hyperosmotic conditions. NIH3T3 cells were incubated for 4.5 h in growth medium or growth medium supplemented with increasing amount of NaCl giving final osmolarity in the range 337 mOsm (isotonic) to 737 mOsm. (A) p53 and histone (house-holding protein) protein expression was detected by western blotting using chemiluminescence detection. Blots represent one out of 17 sets of experiments. (B) p53 expression was determined relative to histone expression from blots shown in (A) and ratios given relative to the value determined in cells incubated with 600 mOsm. Data represent mean values from 16 sets of experiments. *indicates significantly different from the 600 mOsm sample (ANOVA, Student-Newman-Keuls Method). (C) p53 protein expression as function of time following increase in extracellular osmolarity from isotonicity (time zero) to hypertonicity media (500 mOsm, 600 mOsm, 687 mOsm). p53 and histone expression values were determined at time points indicated and the p53/histone ratio at each time point given relative to the expressions ratio at the initial isotonic conditions. Values are mean values from three sets of separate experiments. *indicates significantly increased values compared to isotonic values (Students *t*-test). (D) p53 mRNA expression was in four sets of experiments determined relative to *β*-actin in cells exposed to isotonic or hypertonic media and values given relative to the ratio found in under isotonic (337 mOsm) conditions. No significant variation in p53 mRNA accumulation was detected. (E) p53 phosphorylation (Ser^15^) and histone protein expression were detected by western blotting using chemiluminescence detection. Blots represent one out of five sets of experiments. (F) p53 phosphorylation (pp53) at Ser^15^ was determined in five sets of experiments in the range 537–737 mOsm and given relative to total p53 in the same cell samples at the same tonicity. # and *indicate significantly different from 637 mOsm and 687 mOsm, respectively (ANOVA, Student-Newman-Keuls Method).

Figure1 shows that p53 protein expression respond biphasically to increasing hypertonic stress for 4½ h, that is, expression is low under isotonic (337 mOsm) conditions, increases when the extracellular tonicity is increased from the isotonic 337 mOsm to hypertonic 587–600 mOsm and then decreases as the osmolarity is increased to 737 mOsm (A and B). The p53 expression is given relative to the highest value (600 mOsm) because the p53 band intensity under isotonic conditions was faint and quantification inexact. From Fig.1C it is seen that p53 expression increases with time at 500, 600, 687 mOsm, indicating that the reduction in p53 observed in 687 mOsm does not reflect an oscillating p53 expression. Expression of p53 mRNA does not vary within the range 337–737 mOsm (Fig.1D). Hence, the variation in p53 protein expression seen in Fig.1B must reflect regulation on the protein level. Phosphorylation of p53 is a key event in translocation of p53 to the nucleus and p53-induced activation of apoptosis. We have previously shown that phosphorylation of p53 at Ser^15^ increases with time in NIH3T3 cells following an increase in the extracellular tonicity from isotonic 337 mOsm to 687 mOsm (Friis et al. 2005). Figure1E shows a representative western blot of p53 phosphorylation at Ser^15^ (pp53). It is seen that p53 phosphorylation under isotonic conditions is low but increases transiently with increasing osmolarity. In Fig.1F we compare p53 phosphorylation, shown as the pp53/p53 protein ratio, under conditions of high p53 expression, that is, 537 mOsm and more extreme osmolarities. The highest pp53/p53 ratio is obtained at a tonicity which is higher than the one giving maximal p53 expression.

### Ubiquitination and degradation is decreased at increasing osmotic stress

**Figure 2:**
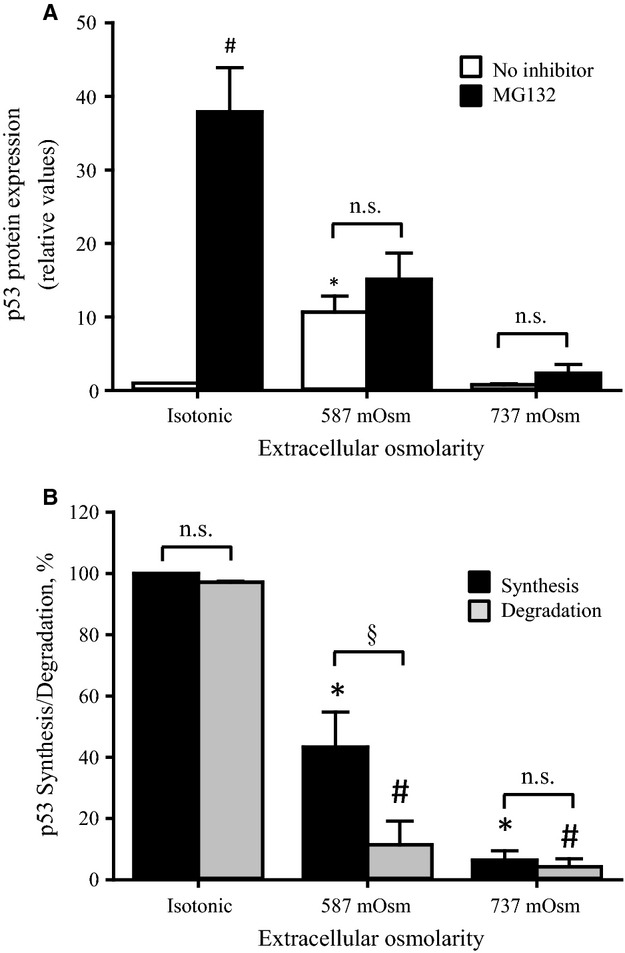
Effect of proteasome inhibition on p53 protein expression in NIH3T3 cells during exposure to isotonic and increasing hyperosmotic conditions. NIH3T3 cells were incubated for 4.5 h in growth medium (isotonic) or growth media supplemented with increasing amount of NaCl (587 mOsm, 737 mOsm). (A) p53 expression was determined in five sets of experiments in the absence (open bars) or presence of 10 *μ*mol/L MG132 (closed bars). Values are given relative to isotonic value with no inhibitor. *indicates significantly increased values compared to the isotonic value. ^#^indicates significantly different from control with no MG132. n.s. indicates no significant effect of MG132 (ANOVA, Student-Newman-Keuls Method). (B) p53 synthesis (closed bars) was for each osmolarity taken as p53 expression in the presence of MG132. p53 degradation (grey bars) was measured as the difference in p53 expression in the presence and absence of MG132. Values are in all cases given in percentage of p53 synthesis under isotonic conditions with no inhibitor and represent mean values of 5 sets of experiments. *indicates significant reduction in p53 synthesis. ^#^indicates significant reduction in p53 degradation. § and n.s. indicate significant or no difference between synthesis and degradation, respectively (ANOVA, Student-Newman-Keuls Method).

In order to analyze the decrease in the protein level of p53 at high osmolarities cells were incubated with the proteasome inhibitor MG132. Figure2A shows that inhibition of proteasome degradation has a strong impact on p53 expression under isotonic conditions whereas the effect is nonsignificant at 587 and 737 mOsm. Using p53 expression in the presence of MG132 as a measure of p53 synthesis and the difference in p53 expression in the presence and absence of MG132 as a measure of degradation, it is seen from Fig. 4B that synthesis as well as degradation decrease with increasing osmolarities. At 587 mOsm the p53 synthesis is significantly threefold larger than degradation, explaining the peak in p53 protein expression seen in Fig.1B.

### p38 phosphorylation as function of increasing osmotic stress

**Figure 3:**
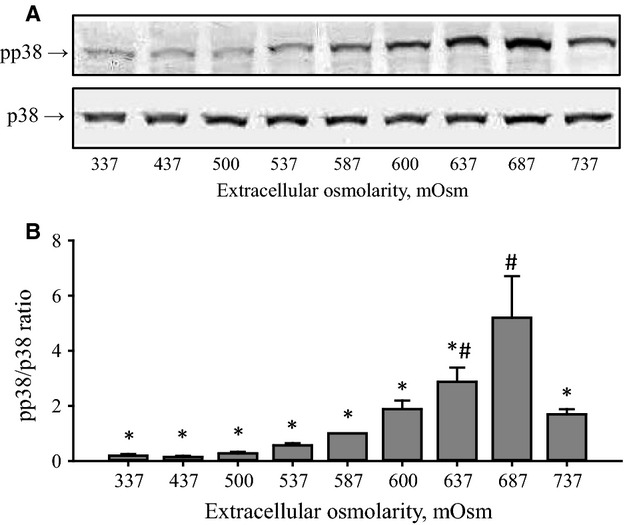
p38 and phosphorylated p38 (pp38) expression in NIH3T3 cells after exposure to increasing hyperosmotic stress. NIH3T3 cells were incubated for 4.5 h in growth medium supplemented with increasing amount of NaCl as indicated in the legend to Fig.1. (A) p38 and pp38 expressions were detected by western blotting using chemo luminescence detection. Blots are representative blots. (B) pp38 (Thr^180^/Tyr^182^) to p38 expressions ratios are given as mean values of four to nine sets of experiments. ^#^indicates significantly increased value compared to the isotonic value. *indicates significantly reduced value compared to the 687 mOsm values (ANOVA, Fisher LSD Method).

It has previously been shown that p38 in its phosphorylated state (pp38) upregulates p53 during osmotic stress by phosphorylation of p53 at Ser^15^ (Friis et al. 2005). Therefore, the expression of p38 and pp38 was measured during increasing osmotic stress. From Fig.3A it is seen that p38 expression is unaffected by the increase in osmolarity, whereas pp38 expression increases in the range 337–687 mOsm and decreases again in the range 687–737 mOsm. The pp38 to p38 expression ratio peaks at 687 mOsm (Fig.3B). Thus, an increase in pp38 correlates with the increase in pp53 (Fig.1E) and with a decrease in p53 degradation.

### MDM2 expression and phosphorylation as function of increasing osmotic stress

**Figure 4:**
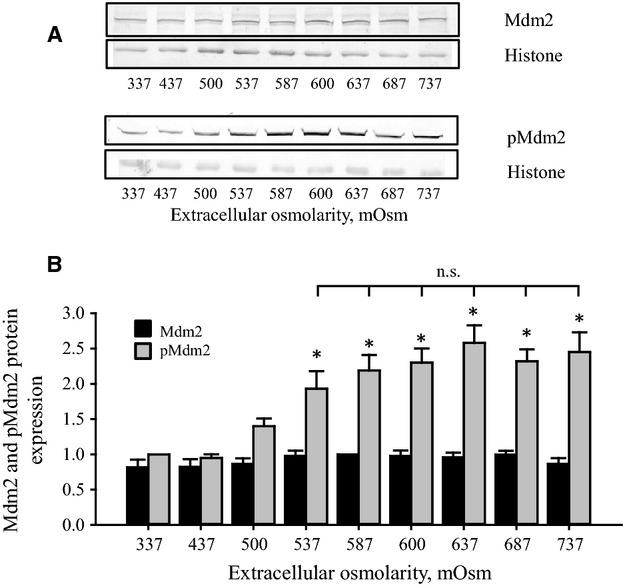
MDM2 and phosphorylated MDM2 (pMDM2) expression in NIH3T3 cells after exposure to increasing hyperosmotic stress. NIH3T3 cells were incubated for 4.5 h in growth medium supplemented with increasing amount of NaCl as indicated in the legend to Fig.1. (A) MDM2 and pMDM2 (Ser^166^) protein expression was detected by western blotting using chemo luminescence detection. Blots are representative blots. (B) MDM2 and pMDM2 expression was determined in at least 12 sets of experiments as outlined in (A) and given relative to the 585 mOsm value (MDM2) of the isotonic value (337 mOsm, pMDM2). *indicates significantly increased value compared to isotonic control. n.s. indicates no significant variation (ANOVA, Student-Newman-Keuls Method). No significant variation in MDM2 expression was detected.

Data in Fig.4A show total MDM2 and phosphorylated MDM2 (pMDM2, Ser^166^) in cells after the incubation with different osmolarities for 4.5 h. It is seen that MDM2 expression is constant in the range from 337 to 737 mOsm, whereas phosphorylation of MDM2 at Ser^166^, which indicates ligase activity, increases up to 537 mOsm and remains constant at higher tonicities (Fig.4B).

### Caspase-3 activity increases as a function of increasing osmotic stress

**Figure 5:**
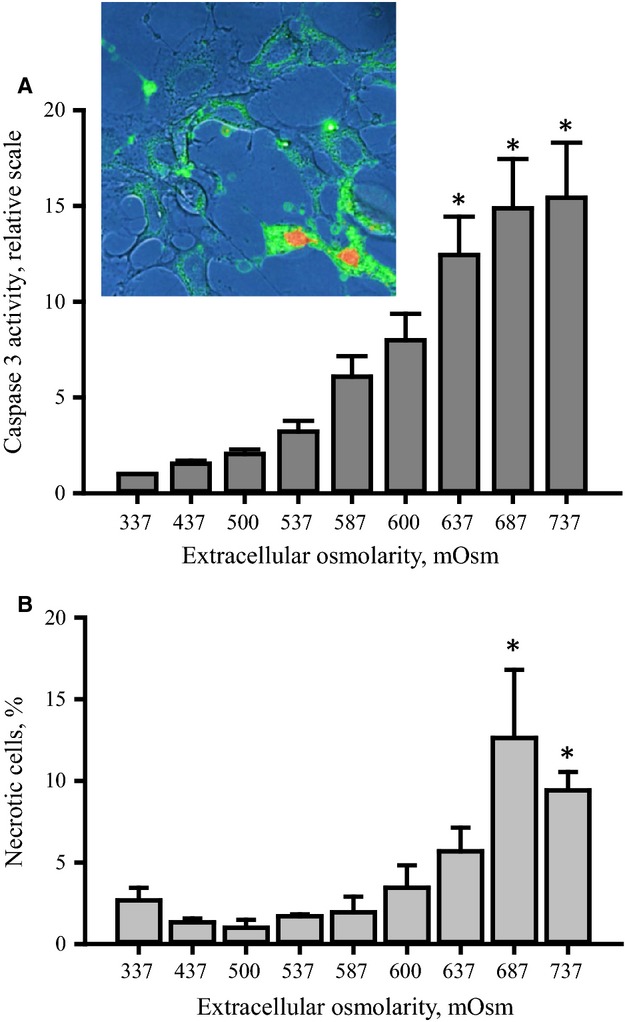
Caspase-3 activity and necrosis in NIH3T3 cells after exposure to increasing hyperosmotic stress. NIH3T3 cells were incubated for 4.5 h in growth medium supplemented with increasing amount of NaCl as indicated in the legend to Fig.1. (A) Caspase-3 activity was determined by a colorimetric assay. Values are given relative to isotonic control and represent means of 11 sets of experiments. (A), insert: Apoptosis and necrosis were detected by annexin V-488 (green, plasma membrane) and propidium iodide (red, nucleus) binding, respectively and confocal laser scanning microscopy on cells exposed to 737 mOsm for 4.5 h. (B) The fraction of necrotic cells was determined from the number of permeable, DAPI-stained cells and the number of acridine orange stained cells as described in Materials and Methods. Values are from three independent sets of experiments. *indicates significantly increased values compared to isotonic values (ANOVA).

Increase in caspase-3 activity is generally taken as an indication of apoptotic progress and we have previously shown that p53 activity is upstream caspase-3 activation under hypertonic conditions (Friis et al. 2005). From Fig.5A it is seen that caspase-3 activity increases as a function of increasing tonicities in the range 337–737 mOsm. To verify apoptosis in NIH3T3 cells exposed to 737 mOsm we detected externalization of phosphatidylserine by binding of annexin V conjugated to green fluorescent FITC dye. The insert in Fig5A shows clear annexin V staining at the plasma membrane, indicating apoptosis. However, the insert also indicates nuclear propidium iodide staining in a few cells, indicating the presence of necrotic cells. Hence, at high osmolarities, that is, 687 and 737 mOsm we have strong activation of caspase-3/apoptotic activity and low/essentially no p53 expression. To quantify the extent of shrinkage-induced necrotic cell death we determined the ratio between permeable, necrotic cells (DAPI staining) and total number of cells (acridine orange staining) following 4½ h exposure to increasing osmotic stress (337 mOsm to 737 mOsm). From Fig.5B it is seen that necrotic cell death is occurring in parallel to apoptosis in cells exposed to extreme tonicities, that is, 687 and 737 mOsm, although to a low degree.

## Discussion

The present data show that p53 protein expression is biphasic in response to an increasing osmolarity, that is, low under isotonic conditions (337 mOsm), dramatically increased following an increase in the extracellular tonicity from 337 to 600 mOsm, and then reduced to isotonic values at 737 mOsm (Fig.1). The low p53 level under isotonic conditions is known to be achieved mainly through MDM2 mediated ubiquitination and proteasomal degradation (Elias et al. 2014). In congruence we find that inhibition of p53 degradation with MG132 under isotonic conditions increases p53 expression in NIH3T3 fibroblasts (Fig.2A).

An increase in p53 expression is seen under many stress conditions and often reflects phosphorylation at Ser^15^ and hence protection of p53 against ubiquitination (Elias et al. 2014). In congruence with previous results (Friis et al. 2005) we find that phosphorylation of p53 at Ser^15^ is increased in NIH3T3 cells after exposure to high osmolarities. The phosphorylation of p53 at Ser^15^ follows a biphasic pattern and the highest pp53 to p53 ratio is obtained at 687 mOsm (Fig.1E). Thus, reduced p53 degradation seen at high osmolarities (see Fig.2B) correlates with increased p53 phosphorylation at Ser^15^. As seen from Fig.2 both synthesis and degradation of p53 decrease at increasing hypotonicity although not to the same degree. The largest difference between synthesis and degradation is seen at 587 mOsm (Fig.2), that is, when we observe the largest p53 protein expression (Fig.1).

We have previously shown that phosphorylation of p38 is increased about eightfold within 180 min in NIH3T3 cells exposed to 687 mOsm compared to isotonic conditions (Friis et al. 2005). Furthermore, inhibition of p38 with SB203580 was shown to reduce the hypertonicity-induced p53 phosphorylation as well as the caspase-3 activation by at least threefold and it was concluded that p38 acts upstream to shrinkage-induced p53 phosphorylation and caspase-3 activation (Friis et al. 2005). In the present report we find that the level of phosphorylated p38 and phosphorylated p53 increases with increasing osmolarities up to 687 mOsm (Figs.1 and 3). Hence, increased p53 phosphorylation and low p53 degradation in NIH3T3 cells at high hypertonic osmolarities correlate with an increased p38 phosphorylation.

MDM2 expression is unchanged during hyperosmotic stress (Fig.4B), which is surprising as an increase p53 normally results in increased expression of MDM2 (Nag et al. 2013). Phosphorylation of MDM2 at Ser^166^, which promotes the E3-ligase activity (Meek and Knippschild 2003) and hence p53 degradation, is increasing in the range 337–537 mOsm, where after it reaches a plateau in the range 537–738 mOsm (Fig.4B). Thus, at high hypertonicity we have a high ligase activity but low p53 degradation (Fig.2B), that is, phosphorylation of MDM2 does not lead to a reduction in p53 expression as the hypertonicity increases from 587 to 737 mOsm presumably because p53 is phosphorylated (Fig.1E) and hence protected. In contrast to the isotonic conditions the present data show that p53 is not degraded by the proteasome at high extracellular osmolarities, as we do not observe a significant difference between p53 expression in the presence and absence of the inhibitor (Fig.2A). From Fig.2B it is seen that p53 synthesis is also decreasing from isotonic to 587 and 737 mOsm, but to a lesser extent than degradation. The largest difference between synthesis and degradation is seen at 587 mOsm, that is, when we observe the largest content in p53 expression. This is in agreement with several reports, which indicate that p53 expression is regulated on the translational level by the ribosomal protein L26 and the transcriptional coactivator nucleolin (Chen et al. 2012). In addition, reduced synthesis of p53 from 337 to 737 mOsm most probably implies general reduction in protein synthesis during hyperosmotic stress as previously described by Kim and Strange (Kim and Strange 2013).

As mentioned in the introduction p53 is upstream caspase-3 when apoptosis is induced via the intrinsic pathway (Vaseva and Moll 2009). We find that caspase-3 activity increases gradually with increasing hypertonicity and becomes significantly increased at osmolarities above 600 mOsm (Fig.5). It is previously demonstrated that hypertonicity increases caspase-3 activity in renal inner medullary epithelial cells (Zhang et al. 2000). However, Dmitrieva and coworkers find that p53 protects the cells from hypertonicity-induced apoptosis at moderate osmolarities (500–600 mOsm) where it causes cell cycle delay, but induces apoptosis at higher osmolarities (700–800 mOsm) (Dmitrieva et al. 2000, 2001). In the present report we find that the caspase-3 activity is unchanged in the range from 337 to about 500 mOsm but increases from 500 to 600 mOsm, which correlates with the increase in p53 expression (compare Fig.1B and Fig.5). However, the increase in caspase-3 activity from 600 to 737 mOsm, takes place under conditions where p53 expression is significantly reduced. From annexin V binding it is verified that the cells at 737 mOsm actually undergo apoptosis. Alternative pathways leading to caspase-3 activation in osmotically shrunken cells have been demonstrated (Hoffmann et al. 2009). In NIH3T3 cells we have previously shown that growth factor receptors become less sensitive under hypertonic conditions resulting in decreased activity of the PI3K/PKB pathway and hence activation of apoptosis (Nielsen et al. 2008). In addition it has been proposed that cell shrinkage leads to increased trafficking of death receptors to the plasma membrane and subsequent activation of caspase-3 (Reinehr et al. 2003; Reinehr and Haussinger 2007). These p53-independent mechanisms could likely be involved in caspase-3 activation in NIH3T3 cells following severe osmotic stress.

## Conclusion

The present data show that the p53 protein level is increased in NIH3T3 cell by hyperosmotic stress until a certain osmolarity and then decreased. Under isotonic conditions synthesis and degradation of the p53 protein are both high and similar in size, which results in low p53 expression. Increasing the extracellular tonicity from isotonic 337 to 737 mOsm results in a decrease in the synthesis as well as the degradation of p53. At 587 mOsm the p53 synthesis exceeds its degradation significantly, which is reflected in high p53 expression, whereas at 737 mOsm degradation is low and balances a low synthesis. A concomitant increase in p38 activity and p53 phosphorylation at Ser^15^ when the extracellular tonicity is increased from 337 to 687 mOsm can to a certain extent explain the reduction in p53 degradation. The reduced synthesis of p53 from 337 to 737 mOsm most probably reflects a general reduction in protein synthesis during hyperosmotic stress. Caspase-3 activity is unchanged at osmolarities up to about 500 mOsm but increases at higher osmolarities. In the range 500–600 mOsm the increase in caspase-3 activity correlates with p53 expression but from 600 mOsm caspase activity is still increasing although p53 expression decreases significantly. This indicates that p53-independent mechanisms contribute to caspase-3 activation after hypertonic conditions in NIH3T3 cells.
